# Mixed-Mode Heat Training: A Practical Alternative for Enhancing Aerobic Capacity in Team Sports

**DOI:** 10.3389/fspor.2020.00071

**Published:** 2020-06-18

**Authors:** Rachel M. Gale, Naroa Etxebarria, Kate L. Pumpa, David B. Pyne

**Affiliations:** ^1^University of Canberra Research Institute for Sport and Exercise, University of Canberra, Bruce, ACT, Australia; ^2^Discipline of Exercise and Sport Science, Faculty of Health, University of Canberra, Bruce, ACT, Australia

**Keywords:** environmental stress, football, fitness, pre-season, running, cycling

## Abstract

**Purpose:** Heat training can be implemented to obtain performance improvements in hot and temperate environments. However, the effectiveness of these interventions for team sports during discrete periods of the season remains uncertain.

**Methods:** We compared the effects of a short pre-season heat training intervention on fitness and thermal tolerance. In a counterbalanced crossover design, eleven state-level male football players undertook 6 × 60 min sessions in HEAT (35°C, 50% RH) and TEMP (18°C, 50% RH) conditions over 12 days. Running performance pre- and post-intervention was assessed via the Yo-Yo Interment Recovery Test Level 1 (YYIR1), and thermal adaptation using a submaximal (4 × 4 min @ 9–13 km·h^−1^) treadmill heat stress test in 35°C, 50% RH.

**Results:** Running distance increased by 9, ±9% in HEAT (standardized mean, ±90% confidence limits) and 13, ±6% in TEMP, the difference in the mean change between conditions was unclear (0.24, ±0.64 standardized mean, ±90% confidence limits). Irrespective of training interventions, there was an order effect indicated by a substantial 476 ± 168 m increase in running distance between the first and final YYIR1 tests. There were trivial to small reductions in heart rate, blood lactate, RPE and thermal sensation after both interventions. Differences in mean core and skin temperature were unclear.

**Conclusions:** Supplementary conditioning sessions in heat and temperate environments undertaken in addition to sports-specific field-based training were effective in enhancing player fitness during the pre-season. However, few clear differences between HEAT and TEMP conditions indicate conditioning in the heat appeared to offer no additional benefit to that of training in temperate conditions.

## Introduction

Physiological, thermoregulatory and performance benefits associated with repeated heat exposures are well-documented (Sawka et al., 1996; Périard et al., 2015). However, heat exposure triggers physiological adaptations that are comparable and often additional to those induced by regular exercise training that, in turn could facilitate performance gains in cooler environmental conditions (Lorenzo et al., 2010; Corbett et al., 2014). If heat acclimation is not the intended (primary) outcome, intermittent heat exposures could offer additional conditioning benefits for team sports. Uncertainty remains on the systematic prescription and scheduling of short-term heat training interventions within a team-sports environment. Given the multifactorial nature of team sports, weekly competition schedules, number of athletes, travel demands, and off-field commitments, coaches and high-performance staff seek pragmatic guidance on how best to tailor traditional heat training regimes to better suit their athletes.

Characteristic thermal adaptations including improved cardiovascular stability, skin blood flow, sweating efficiency, thermal tolerance and reductions in core, and skin temperatures (Sawka et al., 1996; Périard et al., 2015) often develop rapidly. Despite the appeal and apparent ease of implementation, the recommended >7 × 90 min sessions (Périard et al., 2015; Racinais et al., 2015) are often not practically achievable within a team-sport setting. Thus, one key difference between traditional medium-long term and short-term protocols is the total amount of accumulated heat exposure. Total exposure time among short-term regimes involving team-sport players (both consecutive and non-consecutive) ranges from 135 to 324 min (Sunderland et al., 2008; Petersen et al., 2010; Kelly et al., 2016; Gollan et al., 2017; Philp et al., 2017; Duvnjak-Zaknich et al., 2018). These short-term regimes are noticeably shorter than traditional medium- to long-term protocols varying between 450 and 900 min (Nielsen et al., 1993; Lorenzo et al., 2010; Chalmers et al., 2014).

Despite being more practical for teams, non-consecutive heat exposure is likely to limit the opportunity to facilitate thermal adaptation, thus compromising the magnitude of physiological change. Compared to 10 × 30 min bouts of consecutive day heat exposure, 30 min every second day for a total of 10 sessions is less effective in acquiring thermal adaptations than consecutive exposures (Gill and Sleivert, 2001). Similarly, there were few consistent changes in thermal adaptation markers following 8 × 33–47 min high-intensity cycling sessions conducted consecutively and intermittently over 16 days (Duvnjak-Zaknich et al., 2018). Furthermore, eight sessions across 16 days seem equally as effective (as sessions performed consecutively) in improving total work and mean power output in team-sport players during a repeated-sprint cycling heat tolerance test compared to baseline (Duvnjak-Zaknich et al., 2018). It seems non-consecutive, short-term heat training programs have the potential to be integrated into a team-sport setting without compromising sports-specific objectives that might otherwise occur with a consecutive day intervention. However, this may occur at the expense of facilitating complete thermal adaptation.

As game intensities and pressure to win increases, coaches, conditioning and sports science staff seek time efficient and innovate methods for enhancing and/or maintaining performance and player fitness. The challenge is meeting these goals without compromising sports-specific training quality. When heat acclimation is not the priority, heat training has the potential to offer time poor and logistically challenged team sports an alternative, fast-tracked conditioning method. Undertaking conditioning activities in hot environments could offer an additional stress to regular sessions which players would be unaccustomed to. This training intervention could promote physiological and/or performance benefits before and during the regular competitive season. With up to 72 h between exposures being the suggested threshold for offering the potential preservation of adaptive responses (Chalmers et al., 2014), non-consecutive heat exposures may also be effective in reducing residual fatigue while simultaneously providing a stimulus for enhanced performance (Gollan et al., 2017). Cumulative fatigue and insufficient recovery following 5 consecutive days involving 30 min of high-intensity cycling undertaken in conjunction with regular cycling training may have contributed to impaired exercise capacity during subsequent performance in hot conditions (Reeve et al., 2019).

The physiological and performance benefits associated with traditional heat training practices are clearly defined. However, there are questions regarding the scheduling and periodization of heat training regimes among athletes (Casadio et al., 2017), particularly for team-sports. Cycling-based interventions appear to be the preferred mode of supplementary training for a range of team-sports players (Petersen et al., 2010), as practitioners seek to maintain fitness and performance without unnecessarily increasing total running volume and risk of injury (Petersen et al., 2010; Philp et al., 2017). Therefore, a combination of stationary cycling with small bouts of treadmill running, to maintain an element of sports specificity could be an appealing method for gaining supplementary fitness benefits in team-sport players. The purpose of this investigation is to evaluate a pragmatic heat training intervention that would be simple to implement and realistic within a team-sport setting. This information would clarify for coaches whether supplementary conditioning sessions performed in the heat offers fast-tracked fitness and/or performance benefits and thermal adaptation in state-level Australian Rules Football players during pre-season training.

## Materials and Methods

### Participants

Twelve male state-level Australian Rules Football players (age 24.8 ± 5.4 y, body mass 85.1 ± 10.5 kg, height 1.81 ± 0.68 m; mean ± SD) from the same team volunteered for this study. One participant withdrew due to interstate relocation resulting in 11 players completing the study. All players had a sub-elite training history of at least 3 years, with a minimum 4 h on-field training per week during previous pre-seasons. Participants were concurrently engaged in the team's annual 10-weeks pre-season block and continued to maintain normal football training sessions (3 × 90 min sessions·wk^−1^). All participants were advised to avoid any additional training outside of the study and their three football-specific team training sessions. The study was conducted in the late Southern Hemisphere summer and early autumn (~28 ± 1°C; mean daily maximum temperature across study duration). All participants provided written informed consent. The study was approved by the University of Canberra's Human Research Ethics Committee (Project number 15-44).

### Study Design

**Figure 1:**
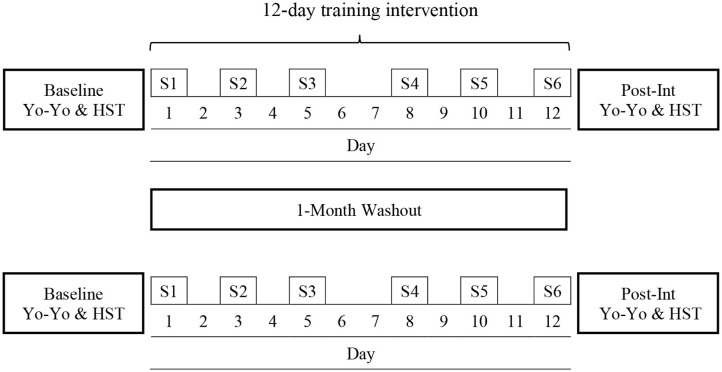
Schematic overview of the counter-balanced crossover study design involving 2 ×12-days training blocks either side of a 1-month washout period. Participants randomly completed the first training block in either HEAT or TEMP conditions. After the washout period participants completed the second training block in the opposite condition to the first trial. YYIR1, Yo-Yo Intermittent Recovery Test Level 1; HST, Heat stress test; S1–S6 refers to the six training sessions.

Participants undertook a counter-balanced crossover design requiring them to complete two separate training interventions (T1 and T2) either side of a 1-month washout period (no intervention). All participants completed 6 × 60 min training sessions (3 sessions·wk^−1^) over 12 days (Figure 1). Participants were randomly assigned to complete T1 in HEAT (*n* = 6; target temperature 35°C, 50% relative humidity [RH]) or TEMP (*n* = 5; target temperature 18°C, 50% RH) conditions. Before and after each 12-days intervention, participants completed a baseline and post-intervention Yo-Yo Intermittent Recovery Test Level 1 (YYIR1) to assess running performance followed by a submaximal Heat Stress Test (HST) to assess thermal adaptation a minimum of 24 h but no more than 72 h after the YYIR1. The YYIR1 and HSTs were performed at the same time of day pre-and-post the 12-days training interventions. After the 1-month wash out period, participants completed the second training intervention (T2) in the opposite condition to the first trial (Figure 1). All training sessions took place in a climate-controlled environmental chamber (Altitude Training Systems, Lidcombe, NSW, Australia).

### Performance and Physiological Testing

The YYIR1 was performed on an indoor running track at the same time of day before and after the 2 × 12-days training interventions in temperate ambient conditions (25 ± 3°C, 54 ± 9% RH) to assess endurance running performance. Briefly, the YYIR1 consists of 2 × 20 m shuttle runs at progressively increasing speeds interspersed with a 10-s active recovery and is controlled via audio tape recording (Bangsbo et al., 2008). Participants ran until they were no longer able to maintain the running speed and/or they failed to meet either end of the 20 m line on two consecutive occasions (Krustrup et al., 2003). The test was performed as a group, all players were familiar with the test and instructions were thoroughly explained to the participants prior to each test. Encouragement was standardized for each Yo-Yo test.

The HST was conducted 24–72 h after the YYIR1 to assess physiological adaptations to the heat training sessions. Participants completed a submaximal HST on a treadmill (TrackMaster TX55, Trackmaster Inc, Newton, KS, United States) in hot conditions (35 ± 2°C, 48 ± 12% RH). This test was adapted from a traditional VO_2max_ protocol (Saunders et al., 2004). After a 5 min warm-up (3 min @ 6 km·h^−1^ and 2 min @ 10 km·h^−1^), participants completed four or five submaximal running stages of 4 min in duration. Each stage was separated by 1 min rest in which the participants were instructed to remain stationary on the treadmill (straddling). Each stage was fixed at 9, 10, 11, 12, and 13 km·h^−1^ (1% gradient), respectively. When blood lactate was <4 mmol/L and Rating of Perceived Exertion (RPE, 6–20 scale) was <16 after the fourth stage participants completed a fifth stage. Eight of the eleven participants completed the fifth running stage at 13 km·h^−1^. The number of stages completed during the initial HST was kept consistent for each individual for the subsequent three HST tests.

During each 1 min rest period between stages, immediately following the cessation of running, heart rate (HR), core temperature (T_c_), skin temperature (T_sk_), blood lactate, RPE and thermal sensation (TS) were recorded to avoid causing disruption to the participant while running. The completion of 4 stages equated to 30 min within the heated environment and 35 min for those who completed five stages. Temperature and RH were monitored using a pocket weather meter (Kestral 4000 Nielsen-Kellerman, PA, USA) fixed to a portable tripod. Participants were permitted a maximum 600 mL of water (actual consumption: HEAT 260 ± 170 mL; TEMP 320 ± 210 mL) to consume *ad libitum* throughout the test.

### Training Interventions

The training interventions involved six sessions each, undertaken every second day (Mon/Wed/Fri) over 12 days in either HEAT (34.4 ± 2.2°C, 56.6 ± 3.1% RH) or TEMP (17.8 ± 0.7°C, 64.7 ± 2.5% RH) conditions. All sessions took place within a climate controlled environmental chamber. After a 5 min warm up, participants completed 5 × 8 min efforts interspersed with 3 min of passive rest. Participants elected to warm up on the Wattbike (Wattbike Ltd, Nottingham, England; air resistance level 1 @ 90 rpm) or treadmill (same ergometer as HST; 3 min @ 6 km·h^−1^ and 2 min @ 10 km·h^−1^). Thereafter, participants alternated between the Wattbike (2.0 W·kg^−1^; individualized per participant) and treadmill (2 km·h^−1^ below final HST speed achieved) for 5 × 8 min efforts (i.e., 3 Wattbike and 2 treadmill efforts). The total session duration was 60 min and all training sessions were completed at the same time of day for each participant. Participants were permitted a maximum 600 mL of room temperature water (actual consumption: HEAT 415 ± 205 mL; TEMP 365 ± 160 mL) to consume throughout the session.

### Physiological Measures

Body mass was recorded before and after the HST and each training session using pre-calibrated scales (FG-150KAL Platform Scales, A&D Weighing Australasia Pty Ltd). Participants were weighed wearing sports shorts only and instructed to towel off as much sweat as possible before the post-exercise measurement. Heart rate was monitored continuously throughout all sessions using Firstbeat Sports analysis software (Firstbeat Technologies Ltd, Jyväskylä, Finland). Four hours before the HST participants ingested a telemetric temperature sensor (CorTemp, HQ Inc., Palmetto, FL, United States) for assessment of T_c_. Skin temperature was assessed on the right, anterior side of the body at the chest, thigh, bicep, and posterior calf (iButton, Maxim Integrated, San Jose, CA, United States). Mean T_sk_ was calculated using the Ramanathan equation (Ramanathan, 1964). A 5 μL capillary blood sample was taken from the fingertip for analysis of blood lactate using a portable Lactate Pro analyzer (Arkray Inc., Kyoto, Japan). HR, T_c_, T_sk_, and blood lactate were assessed at rest and during each 1 min rest period of the HSTs. Heart rate was recorded after each 8 min effort during the environmental training sessions.

### Perceptual Measures

Participant's self-rated their RPE using Borg's 6–20 scale (Borg, 1982) and TS on Young's 8-point (0 unbearably cold-−8 unbearably hot) Likert scale (Young et al., 1987). The RPE and TS scales were thoroughly explained to the participants at the beginning of each YYIR1 and HST to ensure familiarization. RPE and TS were recorded 5 min after completion of the YYIR1 to ensure participants rated the test in its entirety as opposed to the level they exited the test, at rest and during the 1 min rest periods of the HST, and after each 8-min effort during training sessions.

### Statistical Analysis

Descriptive data are presented as mean ± SD. All raw and derived data were collated, checked for outliers and corrected for any errors. Data modeling involved point estimation of YYIR1 test response and thermal adaptation markers (HR, T_c_, T_sk_, RPE, and TS) to heat training, and confidence interval estimates of the uncertainty about the value of these parameters (Curran-Everett, 2009). Markers of thermal adaptations include the 4 × 4 min work intervals only, and do not include the 1 min rest intervals. Values from stage 1 to 5 of the heat stress test were averaged together for a cumulative Baseline and Post-Intervention value. Similarly, HR, RPE, and TS analysis during the six training sessions includes an aggregation of the 5 × 8 min work efforts only and does not include the 5 × 3 min passive rest periods between sets. Data was log-transformed, then back-transformed to obtain changes in means and variation as a percent. Sample size estimation indicated 12 subjects in each condition (HEAT and TEMP) was sufficient to employ a crossover group design to confidently detect a 200 m change in YYIR1 total distance covered (smallest worthwhile change) assuming a typical error of 160 m, and Type I and II errors of 5 and 20%, respectively. Within- and between-participant variability in YYIR1 and thermal markers are reported as the % coefficient of variation (%CV). The typical error of measurement in our laboratory for HST measures are: HR <2%, T_c_ <0.3°C, and T_sk_ <0.3°C. The magnitude of change between the standardized means (ES) was interpreted against the following criteria: <0.19 trivial, 0.2–0.59 small, 0.6–1.19 moderate, 1.2–1.99 large, and >2.0 large (Hopkins et al., 2009). Precision of estimation for all measures (physiological, perceptual and performance) was determined using 90% confidence limits (CL). When the magnitude of the standardized effect crossed the threshold of ±0.2 the change or difference was deemed unclear.

## Results

### Performance Outcomes

**Figure 2:**
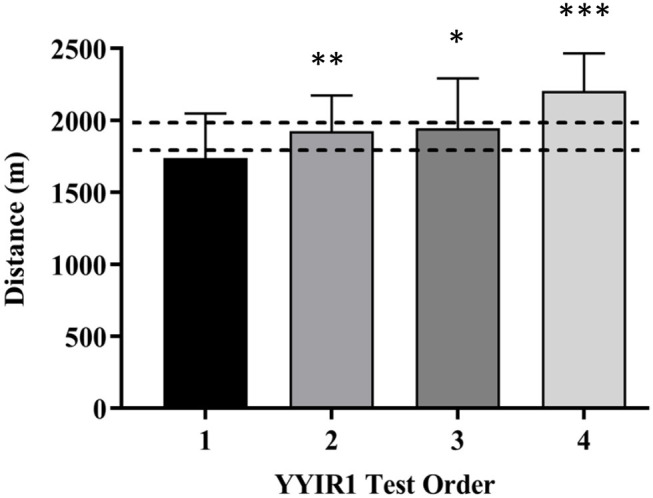
Order effect of changes in Yo-Yo Intermittent Recovery Test Level 1 (YYIR1) running performance relative to the first test. *Small, **Moderate, ***Large effect (Effect Size, ±90% Confidence Limit) relative to test 1. Dotted horizontal zone delineates smallest worthwhile change of 200 m.

Both HEAT and TEMP conditions yielded small to moderate improvements in YYIR1 level and total distance covered after six training sessions (Table 1). Running distance and (consequently) final YYIR1 level improved substantially in both groups: 149 ± 274 m (9, ±9%) in HEAT and 225 ± 156 m (13, ±6%) in TEMP. The differences in mean change between the conditions were unclear for both level and distance covered. Irrespective of the training interventions, there were small to large improvements in YYIR1 running performance indicative of a substantial training effect from the first to last test across the study. Relative to the first YYIR1 test conducted, mean running distance increased by 196 ± 305 m during the second test (0.61, ±0.53 standardized change, ±90% confidence limits), 207 ± 421 m (0.59, ±0.70) during the third test, and 476 ± 168 m (1.31, ±0.30) in the fourth test (Figure 2).

**Table 1:** Yo-Yo Intermittent Recovery Test Level 1 (YYIR1) performance, physiological and perceptual variables between baseline and post-intervention for HEAT and TEMP conditions.

	**HEAT**				**TEMP**				**Difference in change in means ES, ±90% CL**
	**Baseline**	**Post-intervention**	**ES, ±90% CL**	**Magnitude**	**Baseline**	**Post-intervention**	**ES, ±90% CL**	**Magnitude**	
YYIR1 (Level)	18.5 ± 1.0	19.1 ± 0.7	0.49, ±0.42	Small	18.0 ± 1.1	18.9 ± 1.3	0.65, ±0.26	Moderate	0.25, ±0.50; Unclear
Distance (m)	1,924 ± 318	2,073 ± 208	0.43, ±0.43	Small	1,769 ± 350	2,035 ± 386	0.59, ±0.28	Small	0.24, ±0.64; Unclear
Heart rate_max_ (b·min^−1^)	192 ± 9	190 ± 8	−0.25, ±0.23	Small	192 ± 9	191 ± 7	−0.30, ±0.12	Small	−0.08, ±0.35; Unclear
RPE (a.u.)	17 ± 1	18 ± 2	0.48, ±0.51	Small	17 ± 1	18 ± 1	0.83, ±0.56	Moderate	0.37, ±0.79; Unclear
Thermal sensation (a.u.)	7 ± 1	6 ± 1	−0.91, ±0.79	Moderate	6 ± 1	6 ± 1	0.02, ±0.58	Unclear	1.03, ±1.58; Unclear

*Mean ± SD or mean, ±90% confidence limits as specified*.

*YYIR1, Yo-Yo Intermittent Recovery Test Level 1; RPE, rating of perceived exertion; a.u., arbitrary units; ES, effect size; CL, confidence limits*.

### Physiological Outcomes

Mean HR, T_c_, T_sk_, and blood lactate responses to the treadmill HST for HEAT and TEMP conditions are presented in Table 2. There were trivial reductions in submaximal heart rate during the HST in both HEAT and TEMP conditions after the six training sessions. At baseline, during the heat stress test HR increased by 35 ± 9 b·min^−1^ (143 ± 15 to 178 ± 8 b·min^−1^) in HEAT and 37 ± 11 b·min^−1^ (145 ± 13 to 182 ± 8 b·min^−1^) in TEMP. The mean increase in HR was similar between conditions during the post-intervention HST with a progressive increase of 36 ± 9 b·min^−1^ in HEAT and 37 ± 8 b·min^−1^ within TEMP.

**Table 2:** Mean and maximum changes in physiological and perceptual variables during stages 1–5 of the baseline and post-intervention heat stress tests for HEAT and TEMP.

	**HEAT**				**TEMP**				**Difference in change in means ES, ±90% CL**
	**Baseline**	**Post-intervention**	**ES, ±90% CL**	**Magnitude**	**Baseline**	**Post-intervention**	**ES, ±90% CL**	**Magnitude**	
Heart rate (b·min^−1^)	163 ± 14	159 ± 14	−0.19, ±0.03	Trivial	166 ± 15	164 ± 15	−0.13, ±0.09	Trivial	0.07, ±0.10
Heart rate_max_ (b·min^−1^)	177 ± 14	176 ± 14	–	–	182 ± 14	181 ± 15	–	–	Trivial
T_c_ (°C)	37.5 ± 0.3	37.7 ± 0.3	0.40, ±0.18	Small	37.8 ± 0.2	37.4 ± 0.4	−1.84, ±2.06	Unclear	−1.39, ±0.98
T_cmax_ (°C)	37.9 ± 0.3	38.1 ± 0.3	–	–	38.0 ± 0.2	37.9 ± 0.4	–	–	Unclear
T_sk_ (°C)	35.4 ± 0.2	35.6 ± 0.4	0.97, ±1.21	Unclear	35.8 ± 0.3	35.4 ± 0.4	−1.31, ±0.50	Large	−1.51, ±0.48
T_skmax_ (°C)	35.5 ± 0.2	36.0 ± 0.4	–	–	36.1 ± 0.3	35.7 ± 0.4	–	–	Large
Blood lactate (a.u.)	3.1 ± 1.4	2.8 ± 1.3	−0.24, ±0.06	Small	3.6 ± 1.8	3.6 ± 1.5	−0.02, ±0.15	Trivial	0.30, ±0.26
Blood lactate_max_	5.3 ± 1.4	4.8 ± 1.3	–	–	6.2 ± 1.8	5.6 ± 1.5	–	–	Small
RPE (a.u.)	13.3 ± 2.0	12.7 ± 2.2	−0.27, ±0.26	Small	13.7 ± 2.2	13.4 ± 1.7	−0.08, ±0.16	Trivial	0.23, ±0.39
RPE_max_	15.6 ± 2.1	15.2 ± 2.2	–	–	6.1 ± 2.2	15.5 ± 1.7	–	–	Small
Thermal sensation	5.9 ± 0.7	5.6 ± 0.6	−0.31, ±0.04	Small	6.0 ± 0.7	5.9 ± 0.6	−0.10, ±0.19	Trivial	0.25, ±0.27
Thermal sensation_max_	6.6 ± 0.7	6.4 ± 0.6	–	–	6.9 ± 0.7	6.6 ± 0.6	–	–	Small

*Mean and SD, or mean, ±90% confidence limits as specified*.

*T_C_, core temperature; T_sk_, skin temperature; RPE, rating of perceived exertion; a.u., arbitrary units; °C, degrees Celsius; ES, effect size; CL, confidence limits*.

Measures of thermal adaptation, T_c_ and T_sk_ were inconsistent between HEAT and TEMP conditions (Table 2). The six heat training sessions appeared to have little influence on reducing mean T_c_ and T_sk_ within HEAT. Both T_c_ and T_sk_ were ~0.2°C higher during the post-intervention HST than at baseline within HEAT. Conversely, mean exercise T_c_ and T_sk_ were ~0.4°C lower within TEMP after the six training sessions than the baseline trial (Table 2). Given the opposing differences in T_c_ and T_sk_, the difference in T_c_ between conditions was unclear and the uncertainty of T_sk_ within HEAT and large decrease in TEMP resulted in large difference in T_sk_ between the two conditions. There was a greater reduction in blood lactate concentration in HEAT after the training intervention than TEMP (Table 2).

### Perceptual Measures

No substantial differences or changes in post-exercise RPE were observed between conditions after the baseline or post-intervention YYIR1 tests. While self-reported mean RPE was identical in HEAT and TEMP conditions following post-intervention trials, participants in both conditions completed more work (distance covered) at a similar RPE to baseline (Table 1). There were no substantial changes in TS between conditions after the six training sessions. Participants in the HEAT condition reported feeling moderately cooler after the post-intervention YYIR1 test (ES −0.91, ±0.79; Table 1).

Self-reported RPE and TS for HEAT and TEMP conditions during the HST are presented in Table 2. Mean exercise RPE was lower after the post-intervention HST in both conditions but declined further in the HEAT condition. Both HEAT and TEMP conditions displayed similar increases in RPE from stage 1 (10–11, “light”) through to 4 or 5 (15–16, “hard”) of the HST during baseline and post-intervention trials. Perceived TS was lower after HEAT than TEMP training during the post-intervention HST. The six heat training sessions were more effective in reducing self-reported RPE and TS during submaximal exercise than TEMP training (Table 2).

### Training Interventions

**Figure 3:**
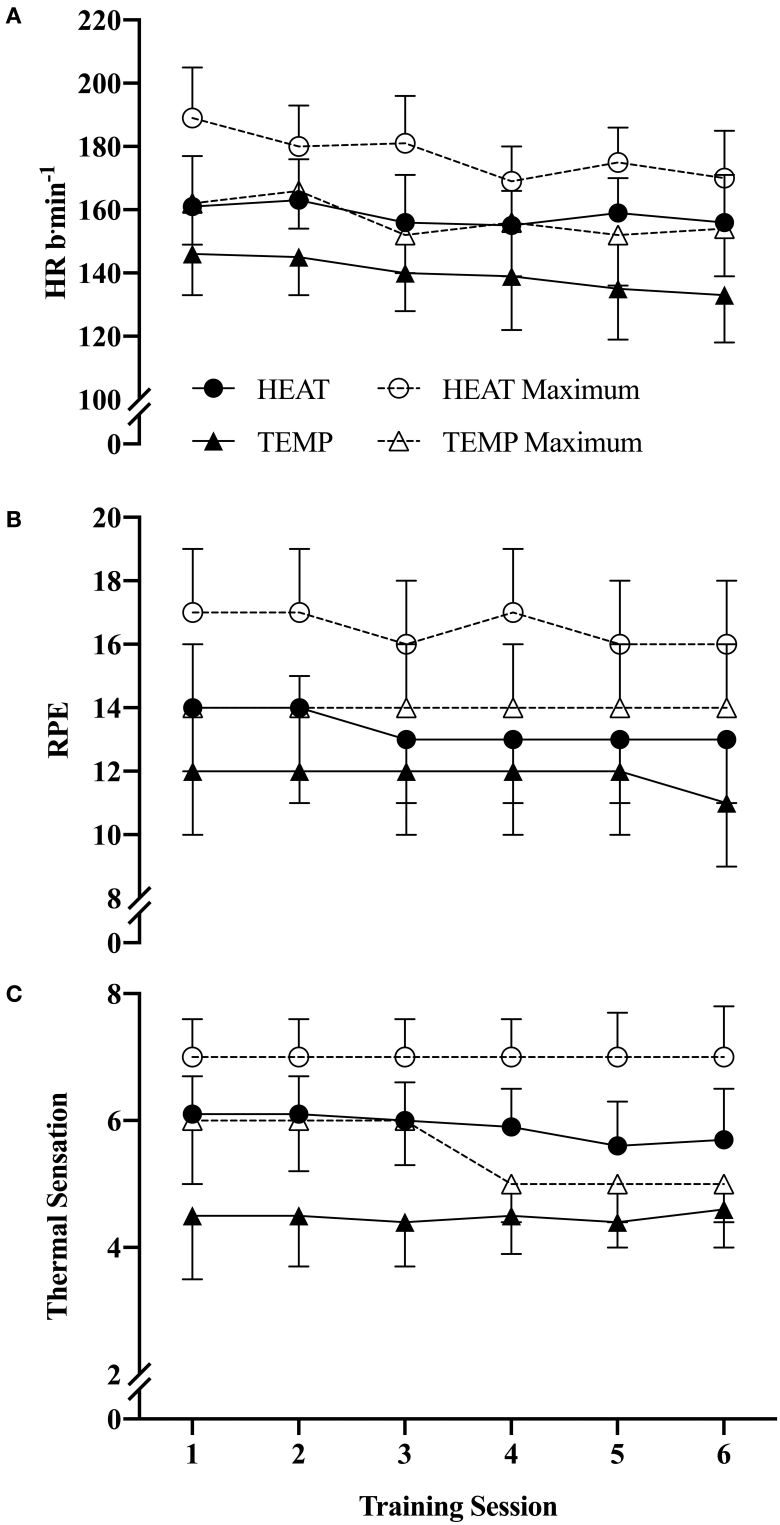
Changes in mean and maximum **(A)** heart rate, **(B)** RPE, and **(C)** thermal sensation after the six training sessions for HEAT and TEMP conditions (mean ± SD). All values are an aggregation of the 5 × 8 min work efforts only and do not included passive rest periods between sets. a.u., arbitrary units.

Reductions in exercising HR were unclear. Exercising HR progressively declined during both HEAT and TEMP training interventions from session one to six by ~5, ±14 and ~11, ±17 b·min^−1^, respectively (mean difference ES −0.45, ±0.99; Figure 3). Reductions in mean exercise HR were observed by the third training session compared to the first in both HEAT and TEMP conditions; however, differences between conditions were unclear (ES −0.13, ±0.49). While there was little change in RPE between the conditions (HEAT −0.8, ±1.2; TEMP −0.7, ±1.0; ES 0.00, ±0.45), perceived TS during exercise was lower in HEAT. No clear changes in TS were observed within the TEMP condition (Figure 3).

## Discussion

When superimposed with sports-specific field training during the pre-season, supplementary conditioning sessions resulted in small improvements in endurance running performance in HEAT and TEMP conditions. However, sessions performed in the heat did not offer any additional performance benefits above those attained by training in temperate conditions. Irrespective of the intervention conditions (HEAT and TEMP) and relative to the first YYIR1 test, there was a 28% improvement in running performance after the final test indicating a substantial training effect. It appears moderately fit team-sport players benefited more from the additional fitness training than training specifically in the heated conditions. The six heat training sessions induced small reductions in blood lactate, RPE and TS during the post-intervention submaximal running test compared to TEMP. There were minimal signs of thermal adaptation in the HEAT condition after the intervention.

While other studies have undertaken non-consecutive heat exposure (Brade et al., 2013; Kelly et al., 2016; Gollan et al., 2017; Duvnjak-Zaknich et al., 2018), to our knowledge, this is the first study to employ a mixed-mode (running and cycling) intervention. In contrast to traditional heat training studies where participants have run/walked and cycled for prolonged periods of time; team sport-players are unlikely to sustain similar continuous efforts. We maintained an element of sports specificity through the inclusion of treadmill running due to the large majority of team sports being running-based. Stationary cycling was incorporated to maintain cardiorespiratory load, reduce musculoskeletal load and avoid risk of injury that may be associated with prolonged treadmill running (Petersen et al., 2010). When matching exercise intensities across the two exercise modes, it is possible the prescribed training intensity was not demanding enough to evoke sufficient thermal stress required for adaptation. Other factors including the non-consecutive nature of the intervention, total exposure time, cumulative heat load and the moderate fitness status of the participants may have also hindered the capacity for clear thermal adaptation to develop in line with more traditional heat training interventions. At the time of the study and with a moderate level fitness, it is unlikely that participants would have been able to sustain higher exercise intensity without causing overtraining and risking injury. The combination of treadmill running and stationary cycling was well-tolerated by the players. This mixed-mode approach offers variety in comparison to the monotony of prolonged treadmill running or stationary cycling, something which team-sport players are not often accustomed to.

In terms of offering a year-round opportunity for teams to engage in heat training, laboratory (artificial) heat exposure provides a useful and relatively accessible means to do so. Although some recommendations are clearer than others, uncertainty remains around the precise combination of training variables (frequency, intensity, duration and number of exposures) for prescribing heat training (Tyler et al., 2016; Daanen et al., 2018). For team-sport players, there appears to be merit in mixed-mode training and performance testing. A small number of studies, including this one had participants complete a cycling intervention (or in our case, cycling and running) and subsequently a running-based performance test (Petersen et al., 2010; Philp et al., 2017). Five consecutive days of 50 min supplementary cycling-based training sessions at 70% heart rate reserve in hot or cool environments yielded small improvements in high-intensity intermittent running abilities in state league Australian Football players (Philp et al., 2017). A study involving cricket players undertook 4 × 30–45 min cycling training sessions coupled with subsequent running-based tests to ensure sports-specificity was maintained (Petersen et al., 2010). The likelihood of team sports undertaking a running-only heat training intervention is not high. Therefore, a combination of mixed-mode testing and training could be an effective means of ensuring performance benefits transfer back to the field or court.

The 12-day heat training intervention in this study provided 360 min of total heat exposure. This offered an additional 110 min beyond previous short-term regimes involving team-sport players accumulating around 250 min of total exposure time (Brade et al., 2013; Philp et al., 2017). This is 36 min extra compared to a recent study utilizing Australian football and soccer players that similarly reported mild thermal adaptation (Duvnjak-Zaknich et al., 2018). Despite the additional heat exposure, differences within but not between conditions align with other reports where changes in intermittent running performance were trivial or unclear following short-term heat training (Petersen et al., 2010; Philp et al., 2017). Likewise, the non-consecutive nature of heat exposure may have contributed toward the variable trivial-small thermal adaptations observed. Although this study set out to develop a pragmatic means of utilizing heat-based training as a conditioning tool for team sports, the protocol implemented was shorter than the traditionally recommended >7 (consecutive) × 90 min sessions. Given the relatively low fitness level among participants, the potential for thermal adaptation to develop was likely obscured by training load-related improvements in fitness. It appears the additional training favored improvements in running performance, as identified through the YYIR1 rather than classical benefits of heat acclimation.

There was a modest disassociation between T_c_, T_sk_ and self-reported TS after the heat training sessions. Participants in the HEAT condition reported feeling cooler after the 12-day intervention despite T_c_ and T_sk_ increasing by ~0.2°C after the post-intervention HST. The participants in the TEMP condition reported a similar TS to baseline, however, T_c_ and T_sk_ were −0.4°C lower than after the post-intervention HST. Given the opposing small (clear) ~0.2°C increase in T_c_ within HEAT and large but unclear −0.4°C decrease within TEMP, the resulting difference in the mean change was large but unclear following the six heat training sessions. These findings are similar to those reported by Petersen et al. (2010) who were also unable to identify clear differences in T_c_ and T_sk_ between the intervention and control groups despite moderate reductions in HR and improved self-reported TS and RPE. It is possible the submaximal HST in this study was not demanding enough at baseline to sufficiently elevate T_c_ and T_sk_ to observe clear changes in the post-intervention trial. The trivial to small reductions in HR, blood lactate, RPE, and TS in both conditions are likely accounted for by general training-induced adaptations associated with the 28% increase in endurance running performance, rather than the environmental stimulus *per se*.

At the time of the study, participants were simultaneously taking part in field-based pre-season training in the late summer months (~28.1 ± 0.6°C; average daily maximum temperature across study duration). To some degree the participants may have been partially acclimatized to the warmer conditions as a result, potentially limiting the magnitude of any thermal adaptations. In conjunction with the participants modest fitness level and short (laboratory) heat exposure, the effects of training were favored over thermal adaptation. While the additional six heat training sessions were successfully implemented into the pre-season training phase of the state-level participants, it is apparent that the nature of the training/heat exposure was inadequate to facilitate heat acclimation. The opportunity to facilitate clear thermal adaptations following this type of heat training intervention may be greater once players have established a higher baseline level of fitness. Another issue is to ensure the workloads between exercise modes and ambient conditions are matched appropriately to elicit marked thermal adaptation. Further, if opting for a non-consecutive day approach, hotter environmental conditions (i.e., 40°C) and additional sessions (i.e., 8–10 sessions across 3 weeks) would be advised. Nonetheless, the six supplementary conditioning sessions could be effectively implemented into routine pre-season training without causing disruption to the players and coaches at this time.

### Conclusion

When integrated with sports-specific training, supplementary conditioning sessions in heat and temperate conditions performed every other day in state-level team-sport players were effective in enhancing player fitness. However, sessions performed in the heat offered no further benefit than being conducted under temperate conditions. As indicated by the 28% increase in endurance running, in a moderately-trained population extra conditioning sessions may suffice during the pre-season preparation as opposed to heat-based training. A mixed-mode (running and cycling) protocol like the one implemented in this study is practically relevant for team sports and was successfully incorporated with routine sports-specific football training without causing disruption.

## Data Availability Statement

The datasets generated for this study are available on request to the corresponding author.

## Ethics Statement

The studies involving human participants were reviewed and approved by University of Canberra Human Research Ethics Committee. The patients/participants provided their written informed consent to participate in this study.

## Author Contributions

RG, NE, KP, and DP contributed to the study design and methodology. RG lead the data collection with the assistance of NE and DP. RG, NE, and DP completed the statistical analysis and interpretation. RG completed the initial article draft and all authors were involved in revising and editing all subsequent drafts. All authors contributed to the article and approved the submitted version.

## Conflict of Interest

The authors declare that the research was conducted in the absence of any commercial or financial relationships that could be construed as a potential conflict of interest.
